# Second Cancers in Patients with Neuroendocrine Tumors

**DOI:** 10.1371/journal.pone.0086414

**Published:** 2013-12-31

**Authors:** Hui-Jen Tsai, Chun-Chieh Wu, Chia-Rung Tsai, Sheng-Fung Lin, Li-Tzong Chen, Jeffrey. S. Chang

**Affiliations:** 1 National Institute of Cancer Research, National Health Research Institutes, Tainan, Taiwan; 2 Department of Internal Medicine, National Cheng Kung University Hospital, Tainan, Taiwan; 3 Department of Internal Medicine, Kaohsiung Medical University Hospital, Kaohsiung Medical University, Kaohsiung, Taiwan; 4 Graduate Institute of Medicine, College of Medicine, Kaohsiung Medical University Hospital, Kaohsiung Medical University, Kaohsiung, Taiwan; 5 Department of Pathology, Kaohsiung Medical University Hospital, Kaohsiung Medical University, Kaohsiung, Taiwan; 6 Institute of Molecular Medicine, National Cheng Kung University, Tainan, Taiwan; Baylor College of Medicine, United States of America

## Abstract

**Background:**

Second cancers have been reported to occur in 10-20% of patients with neuroendocrine tumors (NETs). However, most published studies used data from a single institution or focused only on specific sites of NETs. In addition, most of these studies included second cancers diagnosed concurrently with NETs, making it difficult to assess the temporality and determine the exact incidence of second cancers. In this nationwide population-based study, we used data recorded by the Taiwan Cancer Registry (TCR) to analyze the incidence and distribution of second cancers after the diagnosis of NETs.

**Methods:**

NET cases diagnosed from January 1, 1996 to December 31, 2006 were identified from the TCR. The data on the occurrence of second cancers were ascertained up to December 31, 2008. Standardized incidence ratios (SIRs) of second cancers were calculated based on the cancer incidence rates of the general population. Cox-proportional hazards regression analysis was performed to estimate the hazard ratio (HR) and 95% confidence interval (CI) for the risk of second cancers associated with sex, age, and primary NET sites.

**Results:**

A total of 1,350 newly diagnosed NET cases were identified according to the selection criteria. Among the 1,350 NET patients, 49 (3.63%) developed a second cancer >3 months after the diagnosis of NET. The risk of second cancer following NETs was increased compared to the general population (SIR = 1.48, 95% CI: 1.09-1.96), especially among those diagnosed at age 70 or older (HR = 5.08, 95% CI = 1.69-15.22). There appeared to be no preference of second cancer type according to the primary sites of NETs.

**Conclusions:**

Our study showed that the risk of second cancer following NETs is increased, especially among those diagnosed at age 70 or older. Close monitoring for the occurrence of second cancers after the diagnosis of NETs is warranted.

## Introduction

The occurrence of second cancers could be attributed to the late effect of cancer treatment, genetic susceptibility, such as hereditary cancer-predisposing syndromes, and shared etiologic factors, such as smoking and alcohol [1]. Increased risk of developing second cancers has been reported for various cancers, including testicular, leukemia, lymphoma, head and neck, breast and ovarian cancers [2-8]. In US, 18% of incident cancer cases are second-order or higher-order cancers [1]. Connecticut Tumor Registry reported that the incidence rate of non-simultaneous second cancers was 6.6% among 253,536 cancer patients diagnosed from 1935 to 1982[9].. Among the 57,871 cancer patients treated at the National Cancer Center Hospital of Japan from 1962-1989, the incidence of second cancer was 4% and 59% of second cancers occurred within one year of the first primary cancer [10].. Because the survival of cancer patients has been prolonged due to the improvement in diagnosis and treatments, the risk of developing second cancers is increasingly becoming a serious problem for cancer survivors.

Neuroendocrine tumors (NETs) are neoplasms originating from neuroendocrine cells located throughout the body. Some NETs are associated with familial neuroendocrine syndromes, such as multiple endocrine neoplasia type 1 (MEN-1) and multiple endocrine neoplasia type 2 (MEN-2), while some NETs are sporadic. The cells of NETs can secrete various neuropeptides, which may or may not cause symptoms. Some NET patients are diagnosed due to presentation of symptoms related to “carcinoid syndrome”, whereas some are diagnosed incidentally while undergoing medical examinations for another disease. The behavior and prognosis of NETs are different and may depend on the primary sites and cell differentiation. The median overall survival of NETs is more than 5 years and a longer overall survival is observed for well-differentiated NETs and NETs located in the rectum [11,12]. 

Second cancers have been reported to occur in 10-20% of NET patients [13-16]. However, most of the previous studies used data from a single institution and focused on specific sites of NETs. In addition, most of these studies included second cancers diagnosed concurrently with NETs, making it difficult to assess the temporality and determine the exact incidence of second primary cancers. In this nationwide population-based study, we used data recorded by the Taiwan Cancer Registry (TCR) to analyze the incidence and distribution of second cancers after the diagnosis of NETs. In addition, the risk factors for second cancers after the diagnosis of NETs were evaluated. 

## Materials and Methods

This study was approved by the Research Ethics Committee of the National Health Research Institutes, Taiwan. Data were provided by The Collaboration Center of Health Information Application (CCHIA), Department of Health, Executive Yuan, Taiwan. The CCHIA houses several national databases of Taiwan, including data from the TCR, which can be accessed by researchers through a formal application with a scientific proposal. CCHIA provides de-identified data to the researchers who are only allowed to perform statistical analyses on-site. Researchers have no access to the databases outside the CCHIA. Researchers are only allowed to keep the analytical results (tables or figures) that conform to the CCHIA’s policy (i.e. no individuals can be identified by viewing the analytical results). 

Data used for this study were ascertained from the TCR, which was started in 1979 to monitor and track the changes in rates of cancer incidence and mortality in Taiwan [6]. The TCR captures about 97% of the cancer cases diagnosed in Taiwan [6]. Two indices, the percentage of death certificate only cases (DCO%) and the percentage of morphologically verified cases (MV%), are often used to evaluate the data quality of a cancer registry and the best data quality is indicated by a DCO% of 0 and a MV% of 100 [17]. The DCO% of the TCR improved from 14.2 % in 1996 to 1.2% in 2008 [6]. The MV% ranged from 87.5% in 2002 to 89% in 2008 [6]. These indices showed that the TCR has good data quality that is comparable to that of the other well-established cancer registries around the world [18,19]. 

NET cases diagnosed from January 1, 1996 to December 31, 2006 were identified from the TCR. The morphology (M) codes of the International Classification of Diseases for Oncology, Field Trial Edition (ICD-O-FT) (for those diagnosed from January 1, 1996 to December 31, 2001) or the International Classification of Diseases for Oncology, Third Edition (ICD-O-3) (for those diagnosed after January 1, 2002) were used to identify NET cases. The M codes for NETs included: 8240 (carcinoid tumor), 8241 (enterochromaffin cell carcinoid), 8242 (enterochromaffin-like cell tumors), 8243 (goblet cell carcinoid), 8244 (composite carcinoid), 8245 (adenocarcinoid), 8246 (neuroendocrine carcinoma), 8249 (atypical carcinoid), 8013 (large cell neuroendocrine carcinoma), and 8574 (adenocarcinoma with neuroendocrine differentiation). Those with other cancer diagnosis before and within three months after the diagnosis of NET were excluded. The data on the occurrence of second cancers were ascertained up to December 31, 2008. The observed number of second cancer was compared to the expected number of cancer based on the age- sex-, and site-specific incidence rates of cancer in the general population. The age-, sex-, and site- specific incidence rates of the general population were calculated using the number of new cancer cases by age, sex, and sites recorded in the TCR divided by the age- and sex-specific population reported by the Directorate-General of Budget, Accounting, and Statistics of Taiwan. The expected number of cancer was calculated by multiplying the total person-years accrued from the follow-up of NET patients by the corresponding age-, sex-, and site- specific cancer incidence rates of the general population. The standardized incidence ratios (SIRs) were then calculated by dividing the observed number of second cancers to the expected number of second cancers. The 95% confidence interval (CI) of the SIR was calculated using PAMCOMP version 1.41 [20]. Cox-proportional hazards regression analysis was performed to estimate the hazard ratio (HR) and 95% CI for the risk of second cancers associated with sex, age, and primary NET sites. The Cox-proportional hazards regression analysis was performed using SAS version 9.2 (Cary, NC, USA). 

## Results

### Incidence of second cancers following NETs

A total of 1,350 newly diagnosed NET cases, who did not have another cancer before or within 3 months after the diagnosis of NET, were recorded in the TCR from January 1, 1996 to December 31, 2006. Eight hundred twenty-nine were men (61%) and 521 were women (39%) (Table 1). Among the 1,350 NET patients, 49 (3.63%) developed a second cancer >3 months after the diagnosis of NET. The distributions of sex were similar between NET patients with and without second cancers (*P* = 0.57) (Table 1). NET patients with second cancers were older (mean = 62.3 years old, range: 32-83, 45% diagnosed at ≥ 70 years old) than NET patients without second cancers (mean = 57.1, range: 9-95, 27% diagnosed at ≥ 70 years old) (*P* = 0.06) (Table 1). The primary NET sites of those with and without second cancers were not significantly different; however, a higher proportion of those with second cancers had primary prostate NET (4%) than those without second cancers (0.4%) (Table 1).

**Table 1:** The characteristic of patients with neuroendocrine tumors, Taiwan, 1996-2006.

	Total		without second cancer		with second cancer		
	N	%	N	%	N	%	*P*-value^a^
**Sex**							
Male	829	61.41	797	61.26	32	65.31	0.57
Female	521	38.59	504	38.74	17	34.69	
**Age**							
0-40	208	15.41	204	15.68	4	8.16	0.06
40-50	233	17.26	226	17.37	7	14.29	
50-60	259	19.19	249	19.14	10	20.41	
60-70	273	20.22	267	20.52	6	12.24	
≥70	377	27.93	355	27.29	22	44.90	
**Primary site**							
Rectum	340	25.19	329	25.29	11	22.45	0.12
Lung and bronchus	292	21.63	283	21.79	9	18.37	
Stomach	99	7.33	94	7.23	5	10.20	
Colon	69	5.11	67	5.15	2	4.08	
Pancreas	68	5.04	67	5.15	1	2.04	
Small intestine	66	4.89	63	4.81	3	6.12	
Appendix	54	4.00	50	3.84	4	8.16	
Head and neck^b^	48	3.56	45	3.46	3	6.12	
Liver	22	1.63	22	1.69	0	0.00	
Breast	17	1.26	17	1.31	0	0.00	
Ovary	14	1.04	14	1.08	0	0.00	
Esophagus	11	0.81	11	0.85	0	0.00	
Prostate	7	0.52	5	0.38	2	4.08	
Biliary	7	0.52	7	0.54	0	0.00	
Others^b^	236	17.48	227	17.45	9	18.37	

Abbreviation: N = number

^a^ P-values were calculated using chi-squared test or Fisher’s exact test

^b^ Head and neck includes lip and oral cavity, pharynx, larynx, nasal cavity and paranasal sinuses, middle ear, and major salivary glands; Biliary includes gallbladder and extrahepatic bile duct; Others includes anus, bone, brain, cervix, intracranial gland, kidney, labia majora, mediastinum of the heart, peritoneum, pleura, retroperitoneum, skin, testis, thymus, thyroid, urinary bladder, uterus, vagina, and site undefined

### Risk of developing second cancer in patients with NETs: COX proportional hazards regression

Because rectum was the most common site of NETs among our NET patients, we used rectum as the referent group to analyze the risk of developing second cancers after NETs by different primary sites. Compared to the rectal NET, the risk of developing second cancer was not elevated for the other primary sites, except for prostate NET (univariable HR = 31.71, 95% CI: 6.87-146.35; multivariable HR = 15.21, 95% CI: 3.19-72.44). The risk of second cancer following NETs was not significantly different between women and men. The risk of second cancer was higher for NET patients aged 70 years or older compared to the those younger than 40 years (multivariable HR = 5.08, 95 CI: 1.69-15.22). See Table 2. 

**Table 2:** Risk of second cancer following NETs: univariable and multivariable Cox proportional hazards analysis, Taiwan, 1996-2008.

	Univariable^a^		Multivariable^b^	
	HR	95% CI	HR	95% CI
**Primary tumor site**				
Rectum	Referent		Referent	
Lung and bronchus	1.67	0.69-4.03	1.32	0.54-3.21
Stomach	2.80	0.97-8.08	1.89	0.64-5.58
Colon	1.11	0.25-5.02	1.01	0.22-4.61
Pancreas	0.92	0.12-7.16	1.02	0.13-8.02
Small intestine	2.34	0.65-8.38	1.56	0.43-5.70
Appendix	2.46	0.78-7.44	2.08	0.66-6.58
Head and neck^c^	2.71	0.75-9.72	2.11	0.58-7.65
Prostate	31.71	6.87-146.35	15.21	3.19-72.44
Biliary	-	-	-	-
Others^c^	2.11	0.87-5.12	2.12	0.87-5.17
**Sex**				
Male	Referent		Referent	
Female	0.65	0.36-1.17	0.84	0.46-1.56
**Age**				
Age<40	Referent		Referent	
40<=age<50	1.54	0.45-5.27	1.45	0.42-5.00
50=<age<60	2.65	0.83-8.46	2.60	0.81-8.39
60=<age<70	1.74	0.49-6.19	1.71	0.48-6.14
Age=>70	6.15	2.11-17.88	5.08	1.69-15.22

Abbreviations: CI = confidence interval; HR = hazard ratio

^a^ Hazard ratio and 95% confidence interval were calculated using Cox proportional hazards model.

^b^ Hazard ratio and 95% confidence interval were calculated using Cox proportional hazards model, adjusted for all of the variables in the table.

^c^ Head and neck includes lip and oral cavity, pharynx, larynx, nasal cavity and paranasal sinuses, middle ear, and major salivary glands; Biliary includes gallbladder and extrahepatic bile duct; Others includes anus, bone, brain, cervix, intracranial gland, kidney, labia majora, mediastinum of the heart, peritoneum, pleura, retroperitoneum, skin, testis, thymus, thyroid, urinary bladder, uterus, vagina, and site undefined

### Risk of second cancer in patients with NETs: standardized incidence ratio

The sites of second cancers are listed in Table 3. The most common second cancer developed following NETs was colon cancer (N=6) and lung cancer (N=6). There appeared to be no preference of second cancer type according to the primary site of NETs. Overall, the risk of developing second cancers was higher for NET patients than the general population with a SIR of 1.48 (95% C.I., 1.09-1.96). Compared to the general population, the risk of developing bladder and kidney/renal pelvis/urethra cancer following NETs was higher with a SIR of 3.68 (95% C.I., 1.00-9.43) and 4.48 (95% C.I., 1.22-11.48), respectively. The risk was not significantly elevated for the other types of second cancer. 

**Table 3:** Standardized incidence ratio (SIR) of second cancers following NETs, Taiwan, 1996-2008.

	Observed N			Expected N			Overall			Male			Female		
	**Overall**	**Male**	**Female**	**Overall**	**Male**	**Female**	**SIR** ^a^	**95% CI**		**SIR** ^a^	**95% CI**		**SIR** ^a^	**95% CI**	
**Total cancer**	49	32	17	33.12	22.53	10.60	1.48	1.09-1.96		1.42	0.97-2.01		1.60	0.93-2.51	
**Second cancer type**															
**Colon**	6	6	0	2.71	1.82	0.88	2.22	0.82-4.83		3.29	1.21-7.16		0.00		
**Lung**	6	5	1	4.68	3.64	1.04	1.28	0.47-2.79		1.38	0.45-3.21		0.96	0.02-5.35	
**Breast**	4	1	3	2.12	0.02	2.10	1.89	0.51-4.83		44.80	1.15-253.25		1.43	0.30-4.18	
**Rectum**	3	2	1	2.07	1.45	0.62	1.45	0.30-4.24		1.38	0.17-4.97		1.62	0.04-9.01	
**Liver/intrahepatic bile duct**	5	3	2	4.96	3.78	1.18	1.01	0.33-2.35		0.79	0.16-2.32		1.70	0.21-6.14	
**Prostate**	3	3	0		1.98					1.52	0.31-4.43				
**Bladder**	4	3	1	1.09	0.88	0.21	3.68	1.00-9.43		3.42	0.71-10.00		4.78	0.62-1.37	
**Kidney/renal pelvis/urethra**	4	3	1	0.89	0.57	0.32	4.48	1.22-11.48		5.25	1.08-15.35		3.11	0.79-17.66	
**Esophagus(include EC junction)**	3	2	1	2.76	2.25	0.51	1.09	0.22-3.17		0.89	0.18-3.21		1.96	0.05-10.90	
**Small intestine/pancreas**	2	1	1	0.79	0.55	0.23	2.55	0.31-9.19		1.81	0.05-10.09		4.28	0.11-23.91	
**Cervix/uterus**	2	0	2			1.05							1.90	0.23-6.87	
**Others** ^b^	7	3	4	2.26	1.34	0.91	3.10	1.25-6.40		2.24	0.46-6.53		4.38	1.19-11.22	

Abbreviations: CI = confidence interval; N = number; SIR = standardized incidence ratio

^a^ SIR = observed N/expected N

^b^ Others: soft tissue (N=2), skin (N=1), peritoneum (N=1), thyroid (N=1), and ill-defined (N=2)

## Discussion

In this nation-wide population- and cancer registry-based study, we observed an increased risk of developing second cancers, particularly for urinary tract cancers, among NET patients. Among the 1,350 NET patients diagnosed from 1996 to 2006 in Taiwan, 3.6% developed metachronous second cancers with a SIR of 1.48 (95% CI: 1.09-1.96) compared to the general population. High rates of second primary cancers have been reported in patients with NETs, particularly for gastroenteropancreatic (GEP)-NET with a range of 12-46% [21,22]. However, most of the published studies included synchronous cancers because GET-NETs were frequently diagnosed incidentally during the management for other cancers. In addition, most of the studies were based on a single institution. In population- and registry-based studies that excluded synchronous cancer, high rates of second primary cancer were also observed, although lower than those that included synchronous second cancers. For example, 9.6% developed metachronous cancers among the 8,331 patients with small intestine carcinoid tumors registered in the Surveillance, Epidemiology, and End-Results (SEER) database from 1973 to 2007 [23]. Compared to the general population, those with small intestine carcinoid tumors had an increased risk for the subsequent development of second cancers in the small intestine, liver, prostate, and thyroid [23]. In another study, 5% of the 2,086 colorectal carcinoid patients recorded by SEER from 1973-1996 developed metachronous cancers [24]. For patients with lung carcinoid tumors, 5% developed second cancers 1 year after the diagnosis of primary carcinoid according to the SEER database from 1988 to 2000 [25]. Compared to the general population, those with lung carcinoid tumors had an increased risk for the subsequent development of breast and prostate cancers [25]. Hemminki et al analyzed the Swedish Family-Cancer Database and reported a second cancer rate of 8.2% among 6,646 patients with familial carcinoid tumors of any sites from 1958 to 1998 [26]. Excluding those diagnosed within 1 year after the diagnosis of NETs, the incidence of second cancers was 5.2% [26]. The SIRs for second cancer in any site with more than 1 year of follow-up were significantly greater than one in both men and women [26]. Overall, the incidence rate of second cancer following NET is lower in our study population than those in the US and Sweden, but consistent with previous reports, our results indicated that the risk of second cancers following NETs is increased.

In our study, the risk of urinary tract cancer, including bladder, kidney, renal pelvis, and urethra, was significantly higher for NET patients compared to the general population. In addition, the risk of male breast cancer among men with NETs was higher than the men in the general population. We didn’t see a higher risk of metachronous gastrointestinal tract cancer for NET patients, although more than 50% of NETs were GEP-NETs. According to the previous studies, the sites of second cancers following NETs did not have a clear pattern. The common sites of second cancers following carcinoids in the small intestine and colon were small bowel, liver, prostate, thyroid, lung and urinary tract according to the SEER data [23,24]. Among lung carcinoids, excess risk of breast and prostate cancers was reported [24]. In the Swedish study, increased risk of metachronous second cancer after diagnosis of carcinoid was noticed in small intestine, prostate, skin, endocrine glands, and non-Hodgkin’s lymphoma for men. For women, cancers of upper aerodigestive tract, small intestine, colon, breast, urogenital, melanoma, and leukemia were found to be increased in carcinoid patients [26]. Overall, studies showed that the increased risk of second cancers after NETs may occur in a wide array of body sites. 

The increased risk of second cancer following NETs could be caused by several factors, including genetic, lifestyle, and treatment-related factors. MEN-1 and MEN-2 are the well-known syndromes associated with familial NETs with mutation in the MEN-*1* and *RET* gene, respectively. Patients with familial NETs may be more susceptible to developing another cancer other than NET. In addition to the familial NETs, genetic aberrations in *MEN1*, *ATRX/DAXX*, or mTOR pathway, and *TP53* have also been noticed in sporadic pancreatic NETs [27]. The genetic instability could increase the potential for developing second cancers. Receptors for peptides secreted by neuroendocrine cells, such as secretin, gastrin, bombesin, cholecystokinin, and vasoactive intestinal peptide, have been identified in many cancer types, including cancers of lung, ovarian, thyroid, brain, genitourinary and gastrointestinal tract [21]. Bombesin has been shown to stimulate the growth of breast and pancreatic cancer cells [28,29]. Multiple growth factors, including PDGF, TGF-β, and bFGF, are expressed in the tumor and stroma of GEP-NET and may play a role in the carcinogenesis of second cancers [30-34]. Radiation therapy and chemotherapy have been shown to increase the risk of second cancer. Although there is no evidence of second cancer caused by chemotherapy or radiation therapy for NET patients due to limited case numbers, chemotherapeutic drugs commonly used for NET, such as temozolomide, and doxorubin, were associated with the development of second lymphoma and leukemia in brain tumor and lymphoma patients receiving treatment consisting of these drugs [35-39]. Streptozotocin, an alkylating agent commonly used to treat NETs, also has the potential to induce oncogenesis. Contribution of behavioral and lifestyle factors to second cancers cannot be neglected. Smoking and alcohol are risk factors associated with various cancers, especially aerodigestive tract cancers. The interaction between environmental factors and genetic factors and/or treatment-related factors may promote the carcinogenesis of second cancers after NETs. The interplay between genetic, treatment, and environmental factors in the risk of second cancer after NETs should be further investigated by a large population-based cohort study of patients with NETs. 

Several potential biases must be addressed for the current analysis. First, there might be a concern for the incomplete ascertainment of the cancer cases by the cancer registry (either for the primary NETs or for the second cancers). However, the Taiwan Cancer Registry has a very high coverage for the cancer diagnosed in Taiwan, capturing 97% of the cases. In addition, the registry quality as assessed by MV% and DCO% is comparable to the other well-established cancer registries in the world. Second, diagnostic bias could be a concern due to the closer monitoring of NET patients compared to the general population. In our study, the majority of primary NETs occurred in the gastrointestinal tract. During the follow-up examination of patients with gastrointestinal NETs, one would expect a diagnostic bias to occur due to the increased incidental findings of other gastrointestinal tumors. But to the contrary, although our results indicated an increased risk of second cancers for NET patients, this increase was not over-represented by the gastrointestinal cancers. Rather, the highest SIR for the second cancer was observed for the second cancers occurring in the urinary tract. Therefore, we believe that the diagnostic bias is minimal in our study. 

In summary, our study showed that the risk of second cancer following NETs is increased, especially among those diagnosed at age 70 or older. The second cancer may occur in a wide array of body sites. Close monitoring for the occurrence of second cancers after the diagnosis of NETs is warranted. The contribution of genetic, environmental, and treatment-related factors for second cancers after NETs deserves further exploration.

## References

[1] Travis LB, Demark Wahnefried W, Allan JM, Wood ME, Ng AK (2013) Aetiology, genetics and prevention of secondary neoplasms in adult cancer survivors. Nat Rev Clin Oncol 10: 289-301. doi:10.1038/nrclinonc.2013.41. PubMed: 23529000. 23529000

[2] Travis LB, Curtis RE, Storm H, Hall P, Holowaty E et al. (1997) Risk of second malignant neoplasms among long-term survivors of testicular cancer. J Natl Cancer Inst 89: 1429-1439. doi:10.1093/jnci/89.19.1429. PubMed: 9326912. 9326912

[3] Travis LB, Gospodarowicz M, Curtis RE, Clarke EA, Andersson M et al. (2002) Lung cancer following chemotherapy and radiotherapy for Hodgkin's disease. J Natl Cancer Inst 94: 182-192. doi:10.1093/jnci/94.3.182. PubMed: 11830608. 11830608

[4] Travis LB, Hill DA, Dores GM, Gospodarowicz M, van Leeuwen FE et al. (2003) Breast cancer following radiotherapy and chemotherapy among young women with Hodgkin disease. JAMA 290: 465-475. doi:10.1001/jama.290.4.465. PubMed: 12876089. 12876089

[5] Tajika M, Matsuo K, Ito H, Chihara D, Bhatia V et al. (2013) Risk of second malignancies in patients with gastric marginal zone lymphomas of mucosa associate lymphoid tissue (MALT). J Gastroenterol: ([MedlinePgn:]) PubMed: 23793380. 10.1007/s00535-013-0844-8 23793380

[6] Lee DH, Roh JL, Baek S, Jung JH, Choi SH et al. (2013) Second Cancer Incidence, Risk Factor, and Specific Mortality in Head and Neck Squamous Cell Carcinoma. Otolaryngol Head Neck Surg, 149: 579–586. PubMed: 23820107. 23820107 10.1177/0194599813496373

[7] Curtis RE, Freedman DM, Sherman ME, Fraumeni JF Jr. (2004) Risk of malignant mixed mullerian tumors after tamoxifen therapy for breast cancer. J Natl Cancer Inst 96: 70-74. doi:10.1093/jnci/djh007. PubMed: 14709741. 14709741

[8] Travis LB, Holowaty EJ, Bergfeldt K, Lynch CF, Kohler BA et al. (1999) Risk of leukemia after platinum-based chemotherapy for ovarian cancer. N Engl J Med 340: 351-357. doi:10.1056/NEJM199902043400504. PubMed: 9929525. 9929525

[9] Flannery JT, Boice JD Jr, Devesa SS, Kleinerman RA, Curtis RE et al. (1985) Cancer registration in Connecticut and the study of multiple primary cancers, 1935-82. Natl Cancer Inst Monogr 68: 13-24. PubMed: 4088294. 4088294

[10] Kobayashi Y, Arimoto H, Watanabe S (1991) Occurrence of multiple primary cancer at the National Cancer Center Hospital, 1962-1989. Jpn J Clin Oncol 21: 233-251. PubMed: 1942555. 1942555

[11] Yao JC, Hassan M, Phan A, Dagohoy C, Leary C et al. (2008) One hundred years after "carcinoid": epidemiology of and prognostic factors for neuroendocrine tumors in 35,825 cases in the United States. J Clin Oncol 26: 3063-3072. doi:10.1200/JCO.2007.15.4377. PubMed: 18565894. 18565894

[12] Tsai HJ, Wu CC, Tsai CR, Lin SF, Chen LT et al. (2013) The epidemiology of neuroendocrine tumors in taiwan: a nation-wide cancer registry-based study. PLOS ONE 8: e62487. doi:10.1371/journal.pone.0062487. PubMed: 23614051. 23614051 PMC3632554

[13] Moertel CG, Sauer WG, Dockerty MB, Baggenstoss AH (1961) Life history of the carcinoid tumor of the small intestine. Cancer 14: 901-912. doi:10.1002/1097-0142(196109/10)14:5. PubMed: 13771655. 13771655

[14] Prommegger R, Ensinger C, Steiner P, Sauper T, Profanter C et al. (2004) Neuroendocrine tumors and second primary malignancy--a relationship with clinical impact? Anticancer Res 24: 1049-1051. PubMed: 15154621. 15154621

[15] Perez EA, Koniaris LG, Snell SE, Gutierrez JC, Sumner WE 3rd et al. (2007) 7201 carcinoids: increasing incidence overall and disproportionate mortality in the elderly. World J Surg 31: 1022-1030. doi:10.1007/s00268-005-0774-6. PubMed: 17429568. 17429568

[16] Krausch M, Raffel A, Anlauf M, Schott M, Lehwald N et al. (2013) Secondary malignancy in patients with sporadic neuroendocrine neoplasia. Endocrine 44: 510–516. PubMed: 23494366. 23494366 10.1007/s12020-013-9911-4

[17] Bray F, Parkin DM (2009) Evaluation of data quality in the cancer registry: principles and methods. Part I: comparability, validity and timeliness. Eur J Cancer 45: 747-755. doi:10.1016/j.ejca.2008.11.032. PubMed: 19117750. 19117750

[18] Larsen IK, Småstuen M, Johannesen TB, Langmark F, Parkin DM et al. (2009) Data quality at the Cancer Registry of Norway: an overview of comparability, completeness, validity and timeliness. Eur J Cancer 45: 1218-1231. doi:10.1016/j.ejca.2008.10.037. PubMed: 19091545. 19091545

[19] Shin HR (2008) Global activity of cancer registries and cancer control and cancer incidence statistics in Korea. J Prev Med Public Health 41: 84-91. 18385548

[20] Taeger D, Sun Y, Keil U, Straif K (2000) A stand-alone windows applications for computing exact person-years, standardized mortality ratios and confidence intervals in epidemiological studies. Epidemiology 11: 607-608. doi:10.1097/00001648-200009000-00019. PubMed: 10955416. 10955416

[21] Habal N, Sims C, Bilchik AJ (2000) Gastrointestinal carcinoid tumors and second primary malignancies. J Surg Oncol 75: 310-316. PubMed: 11135275. 11135275 10.1002/1096-9098(200012)75:4<306::aid-jso14>3.0.co;2-3

[22] Niederle MB, Niederle B (2011) Diagnosis and treatment of gastroenteropancreatic neuroendocrine tumors: current data on a prospectively collected, retrospectively analyzed clinical multicenter investigation. Oncologist 16: 602-613. doi:10.1634/theoncologist.2011-0002. PubMed: 21467149. 21467149 PMC3228191

[23] Amin S, Warner RR, Itzkowitz SH, Kim MK (2012) The risk of metachronous cancers in patients with small-intestinal carcinoid tumors: a US population-based study. Endocr Relat Cancer 19: 381-387. doi:10.1530/ERC-11-0392. PubMed: 22454400. 22454400

[24] Tichansky DS, Cagir B, Borrazzo E, Topham A, Palazzo J et al. (2002) Risk of second cancers in patients with colorectal carcinoids. Dis Colon Rectum 45: 91-97. doi:10.1007/s10350-004-6119-y. PubMed: 11786770. 11786770

[25] Cote ML, Wenzlaff AS, Philip PA, Schwartz AG (2006) Secondary cancers after a lung carcinoid primary: a population-based analysis. Lung Cancer 52: 273-279. doi:10.1016/j.lungcan.2006.02.004. PubMed: 16567020. 16567020

[26] Hemminki K, Li X (2001) Familial carcinoid tumors and subsequent cancers: a nation-wide epidemiologic study from Sweden. Int J Cancer 94: 444-448. doi:10.1002/ijc.1473. PubMed: 11745428. 11745428

[27] Jiao Y, Shi C, Edil BH, de Wilde RF, Klimstra DS et al. (2011) DAXX/ATRX, MEN1, and mTOR pathway genes are frequently altered in pancreatic neuroendocrine tumors. Science 331: 1199-1203. doi:10.1126/science.1200609. PubMed: 21252315. 21252315 PMC3144496

[28] Bold RJ, Ishizuka J, Yao CZ, Townsend CM Jr., Thompson JC (1998) Bombesin stimulates in vitro growth of human breast cancer independent of estrogen receptors status. Anticancer Res 18: 4051-4056. PubMed: 9891444. 9891444

[29] Wang QJ, Knezetic JA, Schally AV, Pour PM, Adrian TE (1996) Bombesin may stimulate proliferation of human pancreatic cancer cells through an autocrine pathway. Int J Cancer 68: 528-534. doi:10.1002/(SICI)1097-0215(19961115)68:4. PubMed: 8945626. 8945626

[30] Chaudhry A, Funa K, Oberg K (1993) Expression of growth factor peptides and their receptors in neuroendocrine tumors of the digestive system. Acta Oncol 32: 107-114. doi:10.3109/02841869309083898. PubMed: 8391829. 8391829

[31] Li H, Fredriksson L, Li X, Eriksson U (2003) PDGF-D is a potent transforming and angiogenic growth factor. Oncogene 22: 1501-1510. doi:10.1038/sj.onc.1206223. PubMed: 12629513. 12629513

[32] Wolańska M, Bańkowski E (2006) Fibroblast growth factors (FGF) in human myometrium and uterine leiomyomas in various stages of tumour growth. Biochimie 88: 141-146. doi:10.1016/j.biochi.2005.07.014. PubMed: 16139411. 16139411

[33] Lo J, Hurta RA (2002) Over-expression of K-FGF or bFGF results in altered expression of matrix metalloproteinases: correlations with malignant progression and cellular invasion. Cell Biol Int 26: 319-325. doi:10.1006/cbir.2001.0858. PubMed: 11991661. 11991661

[34] Derynck R, Akhurst RJ, Balmain A (2001) TGF-beta signaling in tumor suppression and cancer progression. Nat Genet 29: 117-129. doi:10.1038/ng1001-117. PubMed: 11586292. 11586292

[35] Momota H, Narita Y, Miyakita Y, Hosono A, Makimoto A et al. (2010) Acute lymphoblastic leukemia after temozolomide treatment for anaplastic astrocytoma in a child with a germline TP53 mutation. Pediatr Blood Cancer 55: 577-579. doi:10.1002/pbc.22542. PubMed: 20658636. 20658636

[36] Neyns B, Cordera S, Joosens E, Pouratian N (2008) Non-Hodgkin's lymphoma in patients with glioma treated with temozolomide. J Clin Oncol 26: 4518-4519. doi:10.1200/JCO.2008.18.8177. PubMed: 18802168. 18802168

[37] Sharma A, Gupta D, Mohanti BK, Thulkar S, Dwary A et al. (2009) Non-Hodgkin lymphoma following temozolomide. Pediatr Blood Cancer 53: 661-662. doi:10.1002/pbc.22090. PubMed: 19533661. 19533661

[38] Fermé C, Mounier N, Casasnovas O, Brice P, Divine M et al. (2006) Long-term results and competing risk analysis of the H89 trial in patients with advanced-stage Hodgkin lymphoma: a study by the Groupe d'Etude des Lymphomes de l'Adulte (GELA). Blood 107: 4636-4642. doi:10.1182/blood-2005-11-4429. PubMed: 16478882. 16478882

[39] Moser EC, Noordijk EM, van Leeuwen FE, Baars JW, Thomas J et al. (2006) Risk of second cancer after treatment of aggressive non-Hodgkin's lymphoma; an EORTC cohort study. Haematologica 91: 1481-1488. PubMed: 17043014. 17043014

